# Tumor-Background Ratio is an effective method to identify tumors and false-positive nodules in indocyanine-green navigation surgery for pediatric liver cancer

**DOI:** 10.3389/fped.2022.875688

**Published:** 2022-07-27

**Authors:** Jun Feng, Hong Qin, Wei Yang, Haiyan Cheng, Jiatong Xu, Jianyu Han, Jianing Mou, Huanmin Wang, Xin Ni

**Affiliations:** ^1^Department of Surgical Oncology, National Center for Children’s Health, Beijing Children’s Hospital, Capital Medical University, Beijing, China; ^2^Department of Pathology, National Center for Children’s Health, Beijing Children’s Hospital, Capital Medical University, Beijing, China; ^3^National Center for Pediatric Cancer Surveillance, National Center for Children’s Health, Beijing Children’s Hospital, Capital Medical University, Beijing, China

**Keywords:** Tumor-Background Ratio, indocyanine green (ICG), children, liver cancer, false positive nodule

## Abstract

**Background:**

Indocyanine green (ICG) navigation surgery has been used for hepatoblastoma (HB) in children but the technique has been reported for using in other childhood liver cancers were rare. This article summarizes the application experience of ICG in HB and other childhood liver cancers in children and explores the role of fluorescence intensity measurement in identifying tumors.

**Methods:**

To summarize the clinical experience of children with liver cancer treated by ICG navigation surgery. The tumor and its surrounding tissue were photographed by near infrared during the operation. The fluorescence intensity of tumors, ICG (+) lesions and the normal liver was measured, and the Tumor-Background Ratio (TBR) was calculated.

**Results:**

A total of 11 children with liver cancer were injected intravenously with ICG 1 day before operation. With the help of ICG fluorescence navigation, there was no residual tumor at the surgical margin for all the children. Total fluorescence was seen in 2 cases, rim fluorescence in 2 cases, and partial fluorescence in 7 cases. 19 ICG false-positive nodules were found on the resection stump or residual liver tissue in 5 cases, and the TBR value of tumors was higher than that of false- positive nodules. 10 children have survived without disease.

**Conclusion:**

ICG navigation surgery is safe and feasible for liver cancer in children, which can enhance the visualization of the tumor during operation and provide more information about the location and boundaries of the tumor. This technique also has limitations, which can be affected by chemotherapy, tumor location, ICG administration regimen, and equipment. TBR is an effective method to identify tumor and non-cancerous lesions.

## Introduction

Liver cancer is one of the most common solid malignant tumors in children, accounting for about 1–4% (1) of childhood cancer, of which hepatoblastoma (HB) is the most common, while hepatocellular carcinoma (HCC), undifferentiated embryonal sarcoma of the liver (UESL) and malignant germ cell tumors of the liver are rare. Surgical resection is the most thorough treatment. The integrity of tumor resection and the residual tumor at the resection stump directly affect the prognosis of children (2).

With the development of the oncology field and the progress of surgical technology, erlying on vision and touch to determine whether a tumor’s boundary has residual tissue to judge whether an operation has been successful or not is out of date and this method is unable to meet the needs of modern accurate resection of malignant tumors. However, indocyanine green (ICG) navigation surgery is expected to solve this problem. This technique was first used in the resection of liver cancer in adults. In recent years, it has been reported that this technique has been applied to HB in children successfully (3–7).

With the increasing experience of using ICG in the operation of HB in children and the continuously updating of supporting equipment, the relationship between tumor fluorescence type and pathological tissue type, the optimal dose, and time of administration are also being discussed. Moreover, the application of ICG in other liver cancer in children also needs further study. This paper summarizes the experience of the application of ICG for children’s HB and other liver cancer and explores the role of fluorescence intensity measurement in identifying tumor types.

## Materials and methods

### Patients

A total of 34 children with liver tumors who underwent surgery in the Department of Oncology, Beijing Children’s Hospital, Capital Medical University from July 2020 to December 2021 were analyzed retrospectively, of which 11 were treated with intraoperative ICG navigation. The clinical information, intraoperative condition, dose, and time of ICG administration, infrared radiographic data of tumor and pathological results of the 11 patients were summarized and analyzed. This study was approved by the Ethics Committee of Beijing Children’s Hospital ([2021]-E-239-R), and informed consent was obtained from the guardians of all children.

### Indocyanine green fluorescence imaging to navigate the surgical process

One day before operation, the 11 patients received intravenous injection of ICG ranging from 0.1 to 0.2 mg/kg. After fully dissociating the liver during the operation, the tumor and the surrounding liver were photographed using real-time near-infrared photography, and the fluorescence imaging degree of the tumor and the surrounding liver was observed. At the same time, the average fluorescence intensity of tumor and the normal liver was measured simultaneously in the same image. Based on the tumor boundary indicated by routine clinical examination, the tumor boundary was marked by fluorescence imaging, and real-time near infrared photography was taken during the operation to determine the scope of surgical resection accurately. After the tumor was removed completely, the residual liver tissue was photographed by near-infrared radiography. The suspicious lesions were observed and the fluorescence intensity was measured before resection. All resected specimens were sent for pathological examination. We learn from the experience of ICG navigation technology in adult cancer resection. In order to eliminate the effects of the dose, time of administration and individual differences in the excretion of ICG on the fluorescence intensity. The fluorescence intensity of normal liver tissue was taken as the background and the tumor and ICG (+) lesions’ Tumor-Background Ratio (TBR) was calculated (8–10). Finally, the fluorescence intensity of the tumor was compared with that of the suspected lesions of ICG (+).

All clinical data for the 11 children were collected, and the general information and clinical features of the children were summarized. According to the pathological results and intraoperative near-infrared radiography data, the experience of preoperative administration of ICG was summarized, and the feasibility, advantages, and disadvantages of ICG navigation surgery in children with liver cancer were analyzed.

Our center used a variety of commercial fluorescence camera systems to complete ICG navigation surgery, including OPTO-CAM2100 (Guangdong Optomedic Technologies, Inc.), FLI-10B (Nanjing Nuoyuan Medical Devices Co., Ltd.), DPM-III-01 (Zhuhai Dipu Medical Technology Co., Ltd.) and the da Vinci Surgical System. Among them, only FLI-10B has the function of measuring fluorescence intensity. OPTO-CAM2100 and DPM-III-01 are fluorescence laparoscopic equipment, which is used to observe tumors *in vitro* through a laparoscope. The da Vinci Surgical System in our center has a near-infrared photography mode, but it cannot measure the fluorescence intensity.

### Data analysis and statistics

SPSS 22.0 statistical software was used to analyze and process the data. The continuous variable of normal distribution is expressed by the mean ± standard deviation, the continuous variable of the non-normal distribution is expressed by the median (lower quartile, upper quartile), and the classified variable is expressed by the number of cases (percentage). Quantitative variables were compared using the Wilcoxon’s rank-sum test. *P*-values < 0.05 were considered to show statistical significance.

## Results

### Clinical features

In this study, there were 11 children, including 8 cases of HB, 1 child with calcified nested stromal-epithelial tumor (CNEST) of the liver, 1 case of yolk sac tumor (YST) of the liver and 1 case of UESL. According to PRE-TEXT staging, there were 6 cases of stage II and 5 cases of stage III (Table 1).

**TABLE 1:** Clinical characteristics of the 11 cases with liver cancer.

No.	Sex	Age (month)	Preoperative diagnosis	PRE-TEXT	Preoperative chemotherapy	Postoperative diagnosis	Postoperative chemotherapy	Follow-up (month)	Prognosis
1	M	8	HB	II	NA	HB	C5VD	17	Alive
2	M	46	HB	III	C5VD	HB	NA	0	Dead (liver failure)
3	M	28	HB	II	C5VD	HB	C5VD	4	Alive
4	M	33	HB	III	C5VD	HB	C5VD	4	Alive
5	M	105	HB	II	NA	CNEST of liver	NA	4	Alive
6	M	41	HB	II	C5V	HB	C5VD	4	Alive
7	M	25	HB	II	C5V	YST of liver	BEP	4	Alive
8	M	36	HB	II	NA	HB	C5V	4	Alive
9	M	32	HB	III	C5VD + ICE	HB	C5VD	3	Alive
10	F	23	HB	III	Plado + ICE	HB	ICE	2	Alive
					IE				
11	F	154	UESL	III	Liposomal doxorubicin + cyclophosphamide	UESL	Liposomal doxorubicin + cyclophosphamide	2	Alive
					TACE(cisplatin) Epirubicin IE				

PRE-TEXT, PRE-Treatment EXTent of tumor; HB, hepatoblastoma; UESL, undifferentiated embryonal sarcoma of liver; YST, yolk sac tumor; CNEST, calcified nested stromal-epithelial tumor; C5V, cisplatin/vincristine/5-FU; C5VD, cisplatin/vincristine/5-FU/doxorubicin; BEP, Bleomycin/Etoposide/Cisplatin; Plado, cisplatin/doxorubicin; ICE, Ifosfamide/carboplatin/etoposide; IE, Ifosfamide/etoposide; TACE, transarterial chemoembolization.

A total of 8 patients received preoperative chemotherapy. Case 7 was clinically diagnosed as HB at the first diagnosis and completed four rounds of C5V chemotherapy. The tumor was significantly reduced after chemotherapy, and the YST was confirmed by pathology after operation. 3 other patients did not receive preoperative chemotherapy. In case 8, a liver mass was found at the age of 12 months. Combined with imaging examination and AFP (−), it was considered as a hepatic hemangioma. After regular reexamination, AFP was found to have increased abruptly at the age of 32 months, so surgical resection was performed considering the possibility of HB.

### Administration time and dose of indocyanine green

None of the children had a history of iodine allergy. ICG was injected intravenously 1 day before operation, and the median time of administration was 24.6 (24.1, 27.7) h. In 9 cases, the dose was 0.1 mg/kg, and the dose was 0.2 mg/kg in 2 cases. No adverse drug reactions were observed after administration (Table 2).

**TABLE 2:** Information of ICG navigation surgery in 11 cases with liver cancer.

NO.	Administration time (h)	Dosage (mg/kg)	Maximum diameter of tumor(cm)	Tumor location (segment)	Surgical approach/device	Fluorescence pattern	TBR	Pathology/necrosis
1	30.6	0.1	4.4	5,6	Open/OPTO-CAM2100	Total		HB (fetal + embryonal)/0
2	24.5	0.2	6.3	5,6	da Vinci Surgical System	Partial		HB (fetal + embryonal + osteoid matrix)/80%
3	26.0	0.2	6.1	3	Open/FLI-10B	Partial		HB (fetal + embryonal)/40%
4	19.4	0.1	9	7,8	Open/FLI-10B	Partial	3.0	HB (fetal + embryonal)/60%
5	24.4	0.1	10.5	5,6	Open/FLI-10B	Rim	4.1	CNEST of liver/0
6	24.1	0.1	6.9	2	Open/DPM-III-01	Partial		HB (fetal + embryonal)/10%
7	24.2	0.1	4.1	5	da Vinci Surgical System	Rim		YST of liver/80%
8	25.8	0.1	3.8	2,4a	Open/FLI-10B	Total	2.7	HB (fetal)/0
9	27.7	0.1	9.8	5,6,8	Open/FLI-10B	Partial	2.0	HB (fetal + embryonal + mesenchymal)/20%
10	29.0	0.1	6.8	4a,7,8	Open/FLI-10B	Partial	2.5	HB (fetal + embryonal)/15%
11	24.0	0.1	13	4a,5,6,7,8	Open/FLI-10B	Partial	2.2	UESL/95%

TBR, Tumor-Background Ratio; HB, hepatoblastoma; CNEST, calcified nested stromal-epithelial tumor; YST, yolk sac tumor; UESL, undifferentiated embryonal sarcoma of liver.

### Surgical methods and fluorescent equipment

All 11 children underwent an non-anatomical hepatectomy. The median diameter of the tumor was 6.8 (4.4, 9.8) cm. The location of the tumor and the involved liver segment are shown in Table 2. Among them, two cases used the da Vinci Surgical System, two cases used a fluorescence laparoscopic camera system to assist open surgery, and seven cases used an open fluorescence camera system.

### Fluorescence imaging

**FIGURE 1:**
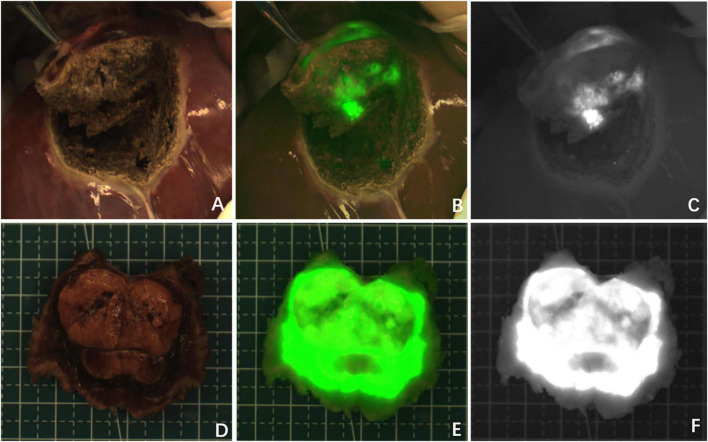
Case 8. Fluorescence can be seen in almost all tumor tissue by near infrared photography *in vivo* **(A–C)** and *in vitro* **(D–F)**. The white-light image, fusion image and fluorescence image are shown in turn from left to right.

**FIGURE 2:**
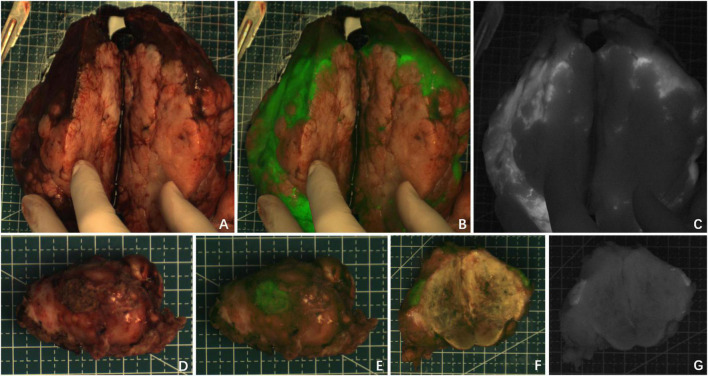
Case 5 and 7. The near infrared photography of the tumor *in vitro* showed rim fluorescence imaging. **(A–C)** Case 5; **(D–G)** Case 7. The white-light image, fusion image and fluorescence image are shown in turn from left to right.

There were two patients who were diagnosed with HB without chemotherapy (Cases 1 and 8) and their tumors showed green fluorescence in almost all tumor tissues under near infrared irradiation (Figure 1). The tumor tissue showed rim fluorescence imaging in two cases (Cases 5 and 7, Figure 2). In case 7, the da Vinci Surgical System was used for surgery. During the operation, it was found that most of the tumors hung outside the liver and only a small part was connected to the liver tissue. There was no fluorescence in the tumor and surrounding liver tissue in the near infrared photography mode. After the tumor was completely resected and irradiated by the open fluorescence camera system *in vitro*, the tumor still had no fluorescence. However, fluorescence imaging could be seen in a small part of the liver tissue connected to the tumor. Paraffin pathology confirmed that the tumor was a yolk sac tumor of the liver, and no tumor cells were found in the fluorescent liver tissue.

**FIGURE 3:**
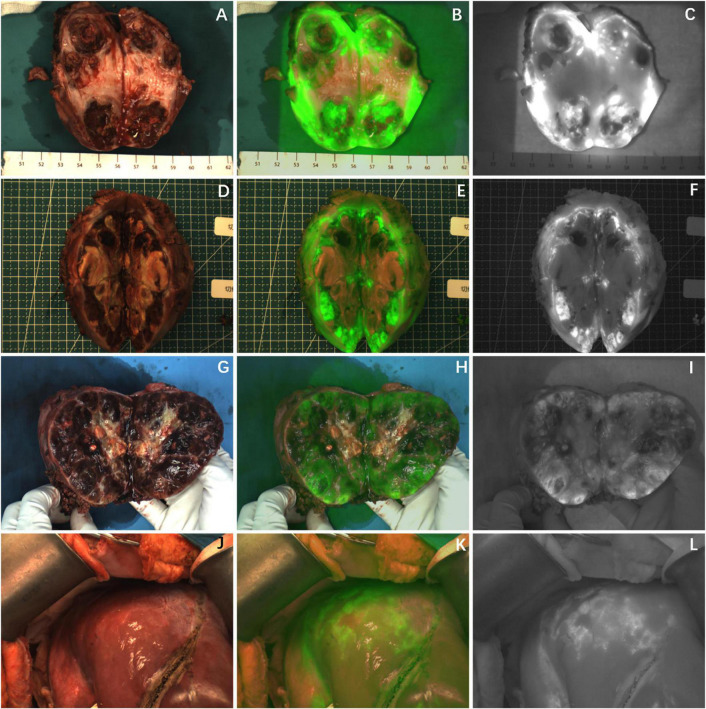
The tumor after chemotherapy showed partial fluorescence imaging, and there was no fluorescence in the area of necrosis or fibrosis. **(A–I)** Fluorescence imaging of 3 cases of HB tumors *in vitro*; **(J–L)** fluorescence imaging of UESL tumor *in vivo*. The white-light image, fusion image and fluorescence image are shown in turn from left to right.

Partial fluorescence imaging could be seen in the tumor tissues of the other seven cases (Figure 3), of which 6 cases were confirmed to be HB after chemotherapy. Only scattered fluorescence imaging was seen in one case, which was confirmed to be UESL after four rounds of chemotherapy and two rounds of transarterial chemoembolization (TACE). The pathological sections of the tumors of the above eight cases after chemotherapy showed different degrees of tumor tissue necrosis and bleeding under the microscope, and the degree of necrosis of UESL tissue with poor fluorescence imaging was the highest (Table 2).

During the operation, it was found that except for the tumor in case 7, which was not recognized by da Vinci near infrared device, the other 10 tumors could be identified by ICG navigation technology. The operator could draw the boundary of the tumor and draw the cutting line according to the difference of fluorescence intensity between the tumor and normal liver tissue. However, not all tumors grow outward. In some cases, the tumors were located inside the liver rather than on the surface, which is a distance from the surface of the liver. It was difficult to determine the cutting line (Table 3). Among them, the depth of the tumor from the surface of the liver in five cases was within 1 cm, and the ICG cutting line of the tumor was consistent with the safe cutting line, and the depth of the intrahepatic part of the tumor from the liver surface in the other six cases was 1.7–5.2 cm. The initial ICG cutting line was not consistent with the safe cutting line, which needed to be combined with palpation and the cutting line should be delineated again according to fluorescence after dissecting the surface liver tissue.

**TABLE 3:** Comparison of preoperative imaging with intraoperative fluorescence imaging.

NO.	Pre-op examination	Tumor depth from liver surface (cm)	The number of the suspected metastasis (CT/MRI)	The number of the suspected metastasis (operation)	ICG pattern of the suspected metastasis	Pathology of the suspected metastasis
1	CT/Ultrasound	1.0	0	0	NA	NA
2	CT/Ultrasound	4.5	0	0	NA	NA
3	CT/Ultrasound	1.7	0	0	NA	NA
4	CT/Ultrasound	2.6	0	1	Negative	No tumor
5	CT/Ultrasound	0	0	1	Positive	No tumor
6	CT/Ultrasound	0	0	0	NA	NA
7	CT/Ultrasound	0	0	0	NA	NA
8	MRI/Ultrasound	0	0	0	NA	NA
9	CT/Ultrasound	4.3	0	0	NA	NA
10	CT/Ultrasound	3.1	0	7	Positive	No tumor
11	CT/Ultrasound	5.2	0	0	NA	NA

CT, Computed Tomography; MRI, Magnetic Resonance Imaging.

**FIGURE 4:**
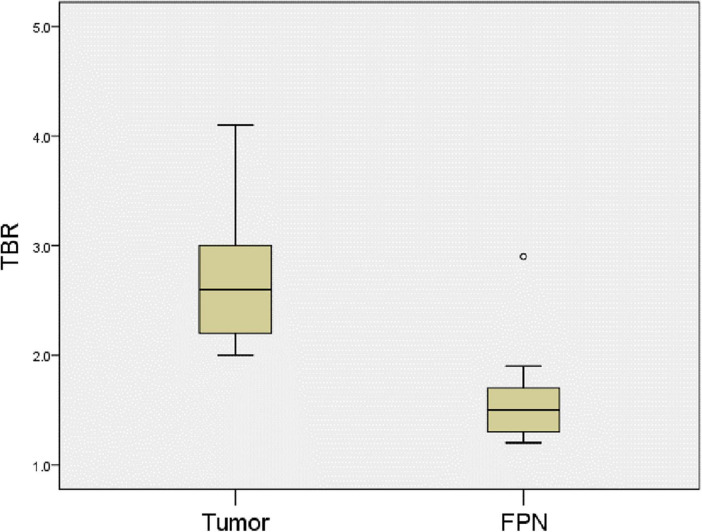
Comparison of TBR values for tumors and false-positive nodules. The TBR values of tumors were higher the false-positive nodules. *P* = 0.0001. FPN, false-positive nodule.

All the 11 children completed CT or MRI before operation, which indicated that there were no other suspected metastatic lesions in the liver except the primary tumor (Table 3). However, one ICG (−) suspected metastasis was found in Case 4 during the operation, and 11 ICG (+) margin and 8 ICG (+) suspected metastasis were found in 5 cases (Case 3, 4, 5, 8, 10) during the operation. These tissues were removed under ICG fluorescence navigation. After confirming that the residual liver was negative by using fluorescence imaging, the resected lesions were sent to pathology for verification. Except for Case 3, the TBR values of ICG (+) lesions at the margin or around the tumor were measured during operation (Table 4), the median TBR values was 1.5 (1.3, 1.7). Pathologically, there were no tumors in these suspicious margin and metastatic lesions, and these ICG (+) lesions were all false-positive nodules under the microscope. The TBR values of tumors were measured in 6 cases (Case 4, 5, 8, 9, 10, 11) during operation (Table 2), the median TBR values was 2.6 (2.2, 3.3). The TBR values of tumors were significantly higher than false-positive nodules, *P* = 0.0001(Figure 4). With the help of the ICG navigation technique, negative margin was achieved in 11 cases, and there was no residual tumor in the margin, which was confirmed by pathology.

**TABLE 4:** Number, TBR value, and histopathology of ICG (+) lesions.

NO.	ICG (+) margin	ICG (+) suspicious metastases	Number	TBR	Pathology
3	√		1		No tumor. 70% fibrous tissues and inflammatory cells, 30% normal liver tissues
4	√		3	1.2	No tumor, normal liver tissue, inflammatory cells
				1.3	
				1.3	
5		√	1	1.8	No tumor, normal liver tissues
8	√		2	1.3	No tumor, normal liver tissues, inflammatory cells, and fibrous tissues
				1.9	
10		√	1 (Left lobe)	1.7	No tumor, normal liver tissues
			1 (Caudate lobe)	2.9	
			5 (Right lobe)	1.3	
	√		5	1.7	No tumor, normal liver tissue, inflammatory cells

TBR, Tumor-Background Ratio; ICG, Indocyanine green.

**FIGURE 5:**
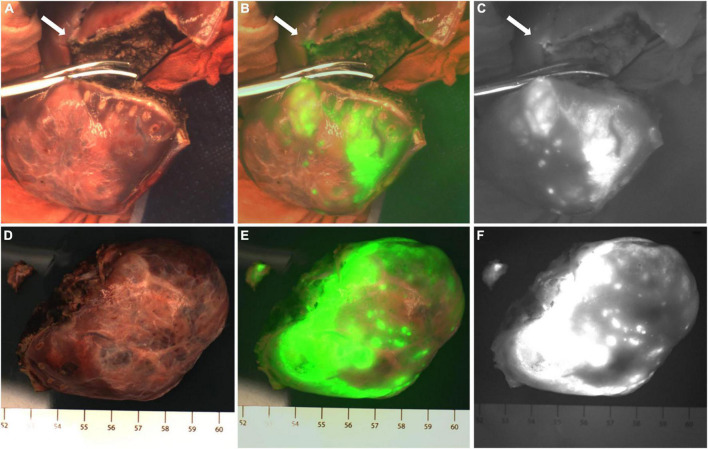
Case 3. Near infrared images of tumors and ICG (+) lesion *in vivo* **(A–C)** and *in vitro* **(D–F)**, with white arrows pointing to ICG (+) lesion. The white-light image, fusion image and fluorescence image are shown in turn from left to right.

In Case 3, after complete resection of the tumor, the residual liver and the resection stump were irradiated with a near-infrared camera system, and an ICG (+) region was found (Figure 5). After surgical resection of this part of the tissue, pathologically showed that about 70% of the tissue was hyperplastic and collagen denatured fibrous tissue, as well as a small number of thick nerve fibers and inflammatory cell infiltration. The remaining 30% was normal liver tissue, and no tumor cells were found.

**FIGURE 6:**
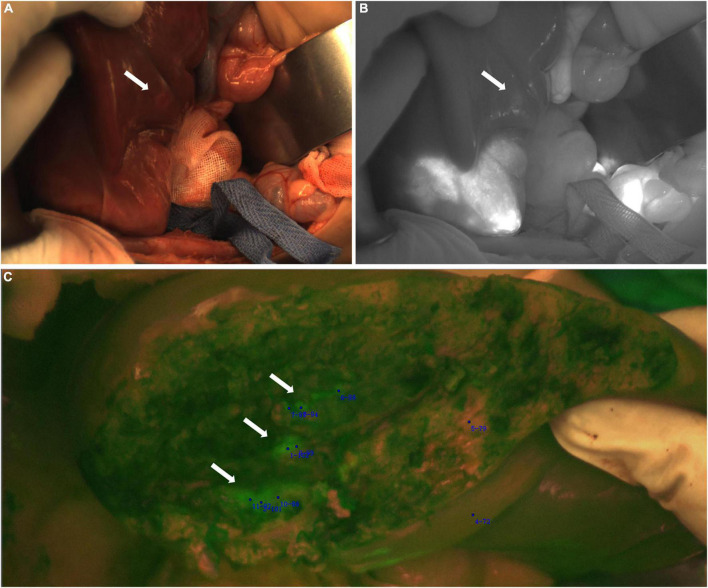
Case 4. Near infrared photography of ICG (−) and ICG (+) lesions during operation. **(A,B)** The white arrow refers to the white light and fluorescence images of suspicious lesions, ICG (−). **(C)** In the fusion image of the resection stump of the residual liver, the white arrow points to 3 ICG (+) regions, respectively.

In Case 4, the TBR value of the tumor was 3.0. Three ICG (+) regions were found in the resection stump of the residual liver (Figure 6C). The TBR values were 1.2, 1.3, and 1.3, respectively. These tissues were removed for pathological examination. We found that they were all normal liver tissue, with a small amount of inflammatory cell infiltration, and no tumor cells. Suspicious metastatic lesions were found about 2 cm away from the tumor (Figures 6A,B). After resection, no tumor cells were found, and all the tissue was either normal liver tissue or intraoperative ICG (−).

**FIGURE 7:**
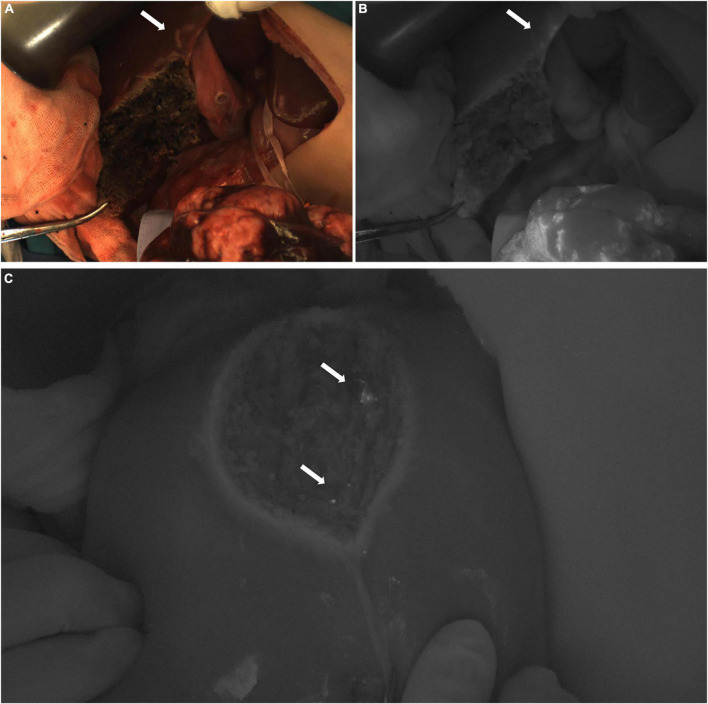
**(A,B)** Case 5. The white light and fluorescence images of the residual liver during the operation, with the white arrow pointing to the ICG (+) lesion away from the tumor 3 cm. **(C)** Case 8. The fluorescence image of the resection stump of the residual liver tissue, with white arrows pointing to two ICG (+) regions, respectively.

In Case 5, the TBR value of the tumor was 4.1. An ICG (+) lesion was found about 3 cm away from the tumor, and the TBR value was 1.8. When the tissue was removed for pathological examination, no tumor cells were found (normal liver tissues, Figures 7A,B).

In Case 8, the TBR value of the tumor was 2.7. Two ICG (+) regions were found in the resection stump of the residual liver tissue, and the TBR values were 1.3 and 1.9, respectively. After removing the tissue for pathological examination, we found normal liver tissue, inflammatory cells, and a little fibrous tissue, but no tumor cells were found under the microscope (Figure 7C).

**FIGURE 8:**
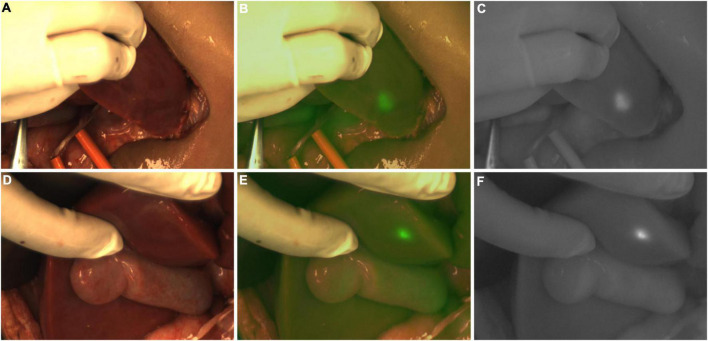
Case 10. Intraoperative near-infrared photography of ICG (+) lesions in the left lobe **(A–C)** and caudate lobe **(D–F)** of the liver. The white-light image, fusion image and fluorescence image are shown in turn from left to right.

**FIGURE 9:**
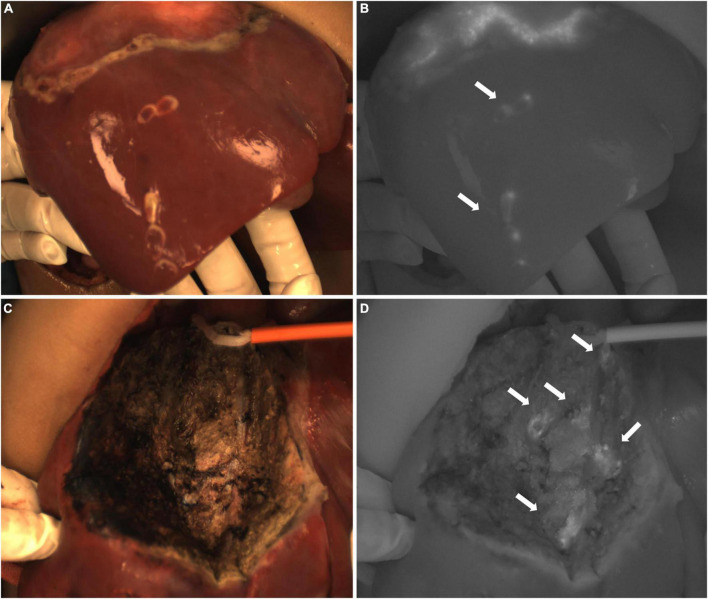
Case 10. **(A,B)** Intraoperative white light and fluorescence images of ICG (+) lesions in the right lobe of the liver, with white arrows pointing to 5 lesions. **(C,D)** The intraoperative white light and fluorescence images of the resection stump of the residual liver tissue, with white arrows pointing to five ICG (+) regions, respectively.

In Case 10, the TBR value of the tumor was 2.5. There were ICG (+) lesions in the left lobe (Figures 8A–C) and caudate lobe (Figures 8D–F) of the liver, and the TBR values were 1.7 and 2.9, respectively. Five ICG (+) lesions were found in the right lobe (Figures 9A,B) of the liver, the TBR values were 1.3, respectively. After removing the tissue for pathological examination, all of it was normal liver tissue and no tumor cells were found under the microscope. In addition, we found 5 ICG (+) regions in the resection stump of the residual liver tissue (Figures 9C,D), the TBR values were 1.7, respectively. When the tissue was removed for pathological examination, we found normal liver tissue and inflammatory cells but no tumor cells.

### Follow-up and prognosis

Among the 11 cases, one patient died of liver failure, and the other 10 cases recovered well without serious complications and continued to receive treatment. The median follow-up time was 4 (2,4) months.

## Discussion

Indocyanine green (ICG) navigation technology is a popular auxiliary surgical technique for tumor fluorescence imaging and has been widely used in the surgical resection of adult liver cancer and other tumors (11, 12). Intravenously injected ICG is actively taken up by healthy hepatocytes and tumor cells, and ICG is taken up by hepatocytes and excreted into bile to be excreted outside the liver after several hours. However, for ICG in tumor cells, the surrounding bile ducts are damaged due to tumor compression and bile excretion is blocked, resulting in ICG retention in the tumor. During surgery, the ICG in the tumor is excited by near-infrared light, causing it to fluoresce, which is captured by the fluorescence camera system, making the tumor visible using fluorescence imaging (3).

In recent years, there have been reports in the literature that ICG navigation surgery can be applied to the surgical treatment of HB in children. This discovery greatly encouraged pediatric oncologists, and it is expected to help remove tumors and avoid residual tumor tissue more completely (13). However, with the continuous updating of the fluorescence camera system and the exploration of ICG medication experience, the practical value, and limitations of ICG navigation surgery for HB and other childhood liver cancers have not been fully elucidated.

In traditional surgery, tumor recognition almost depends on the operator’s experience. The ICG navigation technique was used in 8 cases of HB in this study. The obvious fluorescence imaging of tumors made it possible to visualize the tumor in real time during the operation and improved the operator’s judgment of the location and boundaries of the tumor, especially when using the in da Vinci Surgical System, which lacks tactile and force feedback, ICG showed indispensable value. After resection of the tumor and ICG (+) lesions, there was no fluorescence in the residual liver tissue. It was confirmed by pathology that 8 cases of HB tumors were completely resected and the surgical margin was negative. With the guarantee of this technique, more residual liver tissue is preserved, while tumor residues are avoided, making non-anatomical hepatectomy safer. However, the fluorescence imaging of tumors will also be affected by chemotherapy (5). In this study, 2 cases of HB without chemotherapy showed complete fluorescence imaging of the tumor during the operation, while the other 6 cases of HB showed varying degrees of tumor necrosis or fibrosis due to chemotherapy, and there was no fluorescence in these areas. If these areas are located at the junction of the tumor and normal liver tissue, it will lead to a decrease in the visibility of the tumor location and boundary. Cho et al. also reported 2 cases of HB without fluorescence imaging after chemotherapy, of which 1 case had tumor necrosis of 80%, and the other had mostly calcification and osseous differentiation (14). Therefore, for children with HB undergoing preoperative chemotherapy, this technique still has some limitations.

Three cases of non-HB malignant liver tumors were also included in this study. Case 5, who didn’t have chemotherapy before operation, showed typical rim fluorescence imaging. There was no imaging of the tumor tissue, but the imaging of the liver tissue close to the tumor had obvious contrast, which can help the operator distinguish the boundary of the tumor. The cause of ICG (−) in tumor tissue is related to its pathological features. This is a non-hepatocyte and non-bile duct cell tumor. A nest-like epithelium, spindle cells and myofibroblast stroma can be seen under the microscope, accompanied by varying degrees of calcification and ossification (15). Therefore, the tumor itself does not ingest ICG. In Case 7, the tumor was less connected to the liver tissue, and there was no fluorescence in the da Vinci Surgical System, but we observed the fluorescence of the liver tissue around the tumor on the open device, which was pathologically confirmed as a yolk sac tumor, and 80% of the tumor cells were necrotic. The special pathological type and massive tissue necrosis caused by chemotherapy may be one of the reasons for the lack of fluorescence imaging of the tumor. In Case 11, almost all the tumors were necrotic after chemotherapy and TACE. Also, the location of the tumor was deep, and the fluorescence imaging effect of the tumor during the operation was not good, but it still provided a hint to the operator. After resection of these 3 cases of tumors and ICG (+) lesions, the residual liver tissue showed ICG (−). Confirmed by pathology, these 3 cases of tumors were also completely resected and the surgical margin was negative. Therefore, ICG navigation surgery also has practical value in other liver cancer in children. Although the number of current studies is quite small, it still can be seen that these rare liver tumors can also show a fluorescence effect compared with that of HB.

In this cohort, although preoperative CT or MRI did not indicate other suspicious metastases in the liver, ICG (+) or ICG (−) lesions were found at the margin or around the tumor for 5 children. However, pathologically confirmed that there were no tumors in these suspicious lesions and all ICG (+) lesions were false-positive nodules, it still showed that ICG navigation technology had the potential to detect small metastatic lesions. The reason for the retention of ICG in these nodules is not clear. It has been reported that the histology of false-positive nodules can be characterized by hyperplastic nodules, hyperplastic bile ducts, dysplastic nodules, chronic inflammation, fibrosis, hemorrhage, irregular areas of blood vessels and normal liver parenchyma (16–21). The false-positive nodules found in our study showed chronic inflammatory cell infiltration, normal liver tissue and fibrous tissue under the microscope, which was consistent with the reports in the literature. It is reported in the literature that the false positive rate of ICG is 10 and 20% (22). The number of cases in this study is limited, and the false positive rate of ICG was not calculated. The limited specificity of ICG is one of the causes of false-positive nodules. Operators need to understand this limitation and the risk of extended resection (5). However, the sensitivity of ICG is high. It has been reported that ICG navigation technology can detect nodules with a diameter smaller than 0.1 mm (22, 23). Considering that the goal of tumor surgery is to avoid residue, low specificity may not be an important limiting factor (24). In this study, we found that these false-positive nodules may also be related to the drug administration regimen of ICG in our center.

Hepatocytes take in ICG actively, and most of the literature recommends that the drug be given 72 h before operation, and the recommended dose is 0.5 mg/kg (3, 22). The tumor can be imaged well without an excessive metabolism, and the normal liver tissue also has sufficient time to excrete ICG, so the contrast between them obviously. It is also reported that the dose of 0.3 mg/kg given 2 days before the operation can also achieve good results (20), and it is inferred that the time of administration is proportional to the dose.

In order to enable children to receive more medical monitoring during medication, we tentatively set the administration time of ICG to 1 day before operation. To avoid insufficient excretion of ICG in normal liver tissue due to lack of time, we reduced the dose (0.1–0.2 mg/kg) and measured TBR during operation to make up for the effect of inadequate excretion of ICG in liver tissue. It has been reported that the administration of drugs the day before operation may lead to false-positive nodules due to inadequate excretion of non-cancerous tissue, especially in patients with poor liver function (4). In this study, 5 patients had false-positive nodules, but their tumor imaging was good compared with the surrounding normal liver tissue, so that the operator could clearly observe the location and boundary of the tumor. Compared with adults, children are less likely to have liver cirrhosis and long-term liver insufficiency. Although three of the five patients received chemotherapy before operation, their liver function returned to normal before operation. Therefore, there is little possibility of insufficient excretion of ICG in non-cancerous tissue for children who were given drugs 24 h before operation in this study. Our experience of ICG administration can be used as a clinical reference, especially for children with short preoperative preparation time.

The location and depth of the tumor is also one of the factors affecting fluorescence imaging. Compared with the tumor location of other cases, the tumor location of Case 11 was the deepest. As some of the tumors were far from the surface of the liver, and the tumor cell necrosis rate was as high as 95%, so most of the tumor tissue did not have fluorescence, which is consistent with the reports in the literature. Although ICG can image well in children with liver malignant tumors, when the distance from the tumor to the surface of the liver exceeds 1 cm, the effectiveness of fluorescence imaging decreases significantly (13, 14, 20, 25). Therefore, a deep location of the tumor can lead to low fluorescence or false negative events. In our study, we also find that when the depth of the tumor from the surface of the liver is within 1 cm, the cutting line delineated by fluorescence is consistent with the safe cutting line. When the depth of the tumor from the surface of the liver is greater than 1 cm, the cutting line delineated by fluorescence is not consistent with the safe cutting line, which needs to be redetermined during the operation. Therefore, the depth of the tumor from the surface of the liver should be measured in CT or MRI images before operation, so that the effect of fluorescence imaging can be evaluated in advance. During the operation, the tumor boundary should be judged comprehensively by combining the imaging results, fluorescence imaging and palpation to avoid missing the tumor due to the attenuation of fluorescence.

The difference of equipment is also a factor affecting tumor fluorescence imaging. In case 7, the liver tissue around the tumor had no fluorescence in the da Vinci Surgical System when using fluorescence mode, but showed fluorescence in the open camera system, indicating that the sensitivity of the equipment to capture fluorescence was different. There is a risk of missed diagnosis of small lesions when using less-sensitive devices, resulting in a false negative event. The da Vinci Surgical System used in our center has a single fluorescence mode, and it is impossible to distinguish the normal tissue morphology in the fluorescence mode. During the operation, it needs to switch between the white light mode and the fluorescence mode, which is more tedious to use. When using the open camera system, it is necessary to turn off the operating light in order to make the ICG image clearer. However, the lack of light will affect the appearance of the fine structure of the tumor. We are in the early stages of exploring this technology, so we need to accumulate experience in the use of different equipment.

Although ICG navigation technology is limited by the factors above, it is still the best technique to visualize liver tumors and has great potential for judging the location and size of tumor tissue during operation and assisting surgical resection. When compared to the original equipment, which can only observe the fluorescence imaging of the tumor, with the continuous update of the camera system, some equipment can measure the fluorescence intensity of the tissue quantitatively or semi-quantitatively during the operation. This is the first report of the fluorescence intensity of malignant liver tumors and false-positive nodules in children and the relationship between them. Compared with other camera systems used in the center, the open fluorescence camera system can measure and analyze the fluorescence intensity of different tissues in real time during surgery.

By comparing the TBR values of 6 cases of liver tumors and 18 false-positive nodules, it can be seen of the TBR value of tumors being higher than that of false-positive nodules. This is also consistent with the reported fluorescence intensity of adult HCC and non-cancerous lesions in the literature (17). We speculate that the compression and destruction of the surrounding biliary system by the tumor may be more serious than the benign nodules, but the TBR value of the false-positive nodules in the caudate lobe of Case 10 was higher than that of the tumor. Morita et al. also found that some false-positive nodules have higher fluorescence intensity than HCC (18), but the reason for the increase of the TBR value is not clear. Limited by the number of cases, this study cannot clarify the relationship between fluorescence intensity and pathological type, but this will be a research topic for us in the future. We believe that the measurement of tumor fluorescence intensity will become a hot topic, and it may be possible to predict the pathological type of the tumor by using fluorescence intensity.

Due to the COVID-19 pandemic, the delivery of fluorescence equipment was delayed. Only 11 of 34 children with liver cancer used ICG navigation technology in operation. The longest follow-up time for these patients was 17 months, and the median follow-up time was 4 months. Except for 1 patient who died of postoperative complications, no evidence of tumor recurrence or metastasis was found in the other 10 patients, but the effect of this technique on long-term prognosis could not be fully shown. We will continue to follow up with the children who used ICG navigation technology.

## Conclusion

ICG navigation technology is safe and feasible for liver cancer in children. Whether it is a common HB or a rare liver cancer, ICG navigation technology can enhance the visualization of the tumor during operation and provide more information about the location, boundary and surgical margin of the tumor. However, preoperative chemotherapy, tumor location and depth, ICG administration regimen and the sensitivity of equipment to capture fluorescence all affect the implementation of this technique, which may lead to false positive or false negative events. It is worth noting that the determination of fluorescence intensity adds a quantified analytical method to this technique, and future studies may establish a relationship between TBR values of tumor and pathological types. Therefore, ICG navigation technology still has great potential in the operation of liver cancer in children.

## Data availability statement

The original contributions presented in the study are included in the article/supplementary material, further inquiries can be directed to the corresponding author/s.

## Ethics statement

The studies involving human participants were reviewed and approved by the Ethics Committee of Beijing Children’s Hospital, Capital Medical University. Written informed consent to participate in this study was provided by the participants or their legal guardian/next of kin.

## Author contributions

JF drafted the study design and the manuscript and analyzed the results. HW and HQ made substantial contributions to the conception of the study and revised it critically. XN analyzed the work and revised it critically. WY, HC, JX, JH, and JM participated in the data acquisition and made substantial contributions to the data interpretation. All authors made an important scientific contribution to the study and was thoroughly familiar with the primary data, approved the version to be published and all agreed to be accountable for all aspects of the work in ensuring that questions related to the accuracy and integrity of any part of the work are appropriately investigated and resolved.

## Abbreviations

ICG: Indocyanine Green
HB: Hepatoblastoma
TBR: Tumor-Background Ratio
HCC: Hepatocellular Carcinoma
UESL: Undifferentiated Embryonal Sarcoma of Liver
PRE-TEXT: PRE-Treatment EXTent of tumor
CNEST: Calcified Nested Stromal-Epithelial Tumor
YST: Yolk Sac Tumor
AFP: Alpha-Fetoprotein
TACE: Transarterial Chemoembolization
C5V: Cisplatin/Vincristine/5-FU
C5VD: Cisplatin/Vincristine/5-FU/Doxorubicin
BEP: Bleomycin/Etoposide/Cisplatin
PLADO: Cisplatin/Doxorubicin
ICE: Ifosfamide/Carboplatin/Etoposide
IE: Ifosfamide/Etoposide
FPN: false-positive nodule
CT: Computed Tomography
MRI: Magnetic Resonance Imaging.
